# Antibiotic resistance and virulence characteristics of *Vibrio vulnificus* isolated from Ningbo, China

**DOI:** 10.3389/fmicb.2024.1459466

**Published:** 2024-08-05

**Authors:** Xiaomin Xu, Shanyan Liang, Xin Li, Wenjin Hu, Xi Li, Liusheng Lei, Huai Lin

**Affiliations:** ^1^Department of Hospital Infection Management, Ningbo No.2 Hospital, Ningbo, China; ^2^State Key Laboratory of Pollution Control and Resource Reuse, School of the Environment, Nanjing University, Nanjing, China; ^3^Shenzhen Research Institute of Nanjing University, Shenzhen, China

**Keywords:** *Vibrio vulnificus*, antibiotic resistance genes, virulence factors, molecular characteristics, China

## Abstract

**Background:**

*Vibrio vulnificus* (*V. vulnificus*) is a deadly opportunistic human pathogen with high mortality worldwide. Notably, climate warming is likely to expand its geographical range and increase the infection risk for individuals in coastal regions. However, due to the absence of comprehensive surveillance systems, the emergence and characteristics of clinical *V. vulnificus* isolates remain poorly understood in China.

**Methods:**

In this study, we investigate antibiotic resistance, virulence including serum resistance, and hemolytic ability, as well as molecular characteristics of 21 *V. vulnificus* isolates collected from patients in Ningbo, China.

**Results and discussion:**

The results indicate that all isolates have been identified as potential virulent *vcg* C type, with the majority (16 of 21) classified as *16S rRNA* B type. Furthermore, these isolates exhibit a high level of antibiotic resistance, with 66.7% resistance to more than three antibiotics and 61.9% possessing a multiple antibiotic resistance (MAR) index exceeding 0.2. In terms of virulence, most isolates were categorized as grade 1 in serum resistance, with one strain, S12, demonstrating intermediate sensitivity in serum resistance, belonging to grade 3. Whole genome analysis disclosed the profiles of antibiotic resistance genes (ARGs) and virulence factors (VFs) in these strains. The strains share substantial VF genes associated with adherence, iron uptake, antiphagocytosis, toxin, and motility. In particular, key VFs such as capsule (CPS), lipopolysaccharide (LPS), and multifunctional autoprocessing repeats-in-toxin (MARTX) are prevalent in all isolates. Specifically, S12 possesses a notably high number of VF genes (672), which potentially explains its higher virulence. Additionally, these strains shared six ARGs, namely, *PBP3*, *adeF*, var*G*, *parE*, and *CRP*, which likely determine their antibiotic resistance phenotype.

**Conclusion:**

Overall, our study provides valuable baseline information for clinical tracking, prevention, control, and treatment of *V. vulnificus* infections.

## 1 Introduction

*Vibrio vulnificus* is a gram-negative bacterium that typically occurs in estuarine and marine environments, frequently contaminating bivalves worldwide (Baker-Austin et al., 2018). It infects humans through the consumption of raw contaminated seafood or contact with contaminated seawater, causing gastroenteritis or septicemia, especially in individuals with underlying chronic diseases or immunocompromised systems (Pan et al., 2013). The global incidence rate varies, with an estimated 40 cases of *V. vulnificus* per year in the United States, 425 cases in Japan, and 37–88 cases in South Korea (Osaka et al., 2004; Gulig et al., 2005). Notably, in Taiwan, infection cases ranged from 13 to 26 per year between 1996 and 2000 (Hsueh et al., 2004; Oh et al., 2023). Importantly, *V. vulnificus* accounts for approximately 95% of all seafood-associated illnesses, with a mortality rate of approximately 50% (Jones and Oliver, 2009). However, although there are reports of *V. vulnificus* in Guangdong and Zhejiang in China, the incidence of cases remains limited due to a lack of comprehensive surveillance systems for *Vibrio* infection in China (Cai et al., 2021; Wang et al., 2023). Furthermore, with the effects of global warming, the geographical extent of *V. vulnificus* has greatly expanded, leading to increasing infection rates (Vezzulli et al., 2016). Therefore, it is crucial to collect clinically infected strains and analyze their characteristics in China to gain insights into clinical manifestations, treatment options, and prevention strategies for *V. vulnificus*.

Virulence factor (VF) and antibiotic resistance significantly contribute to the infection capability of *V. vulnificus*. Strains carrying various virulence factors, such as capsular polysaccharide (CPS), lipopolysaccharide (LPS), iron acquisition systems, flagella, pili, hemolysin/cytolysin, metalloprotease, and repeats-in-toxin (RTX) are critical for their adherence, invasion, survival, and infection. These VFs play vital roles in their pathogenicity. Therefore, VF-encoding genes are often used to distinguish between virulent and non-virulent strains. For instance, RTX-positive strains have been reported to exhibit greater virulence than RTX-negative strains (Gulig et al., 2005; Blanke et al., 2012). There is a positive correlation between the presence and amount of CPS and the quantitative virulence of the virulent isolates (Yoshida et al., 1985; Strom and Paranjpye, 2000). Clinical *V. vulnificus* strains frequently possess RTX- and CPS-encoding genes, indicating their role in infections (Lopez-Perez et al., 2019; López-Pérez et al., 2021). However, there is no clear consensus on whether there exists a particular VF that determines virulence or distinguishes pathogenic versus non-pathogenic *V. vulnificus* strains (Strom and Paranjpye, 2000). Therefore, analyzing the VF gene profiles of infected isolates is important to elucidate their underlying virulence potentials. Additionally, antibiotic resistance is another key factor associated *V. vulnificus* infection. Although *V. vulnificus* is generally susceptible to most antibiotics (Elmahdi et al., 2016), there has been an emergence of resistance. Specifically, *V. vulnificus* demonstrates 100% resistance to ampicillin and cefazolin (Zanetti et al., 2001; Sony et al., 2021). Moreover, the reports indicate an increasing antibiotic resistance of *V. vulnificus* to commonly used antibiotics such as cephalosporin and tetracyclines (Baker-Austin et al., 2009). Numerous strains have exhibited resistance to eight or more antibiotics (Elmahdi et al., 2016). The increasing resistance is attributed to the misuse and excessive use of antibiotics, leading to a significant rise in *Vibrio* antibiotic resistance that is expected to continue. Hence, there is an urgent need to monitor the antibiotic resistance of clinical *V. vulnificus* to provide guidance for antibiotic treatment. Moreover, analyzing antibiotic resistance genes in these opportunistic pathogens is crucial as these genes not only determine the antibiotic phenotype but can also acquire antibiotic resistance through horizontal transfer. Next-generation sequencing, along with genome analysis, provides insights into the genomic characteristics of these VF genes and ARGs, increasingly being used in the study of *V. vulnificus* (Oh et al., 2023). Therefore, the characterization of *V. vulnificus* from both perspectives of VF genes and ARGs using genome analysis is indispensable for effective infection tracking.

To achieve these goals, the present study seeks to analyze virulence and antibiotic resistance features of clinically isolated *V. vulnificus* strains in Ningbo, China. Furthermore, through genome analysis, we aim to further characterize the VF genes and ARGs present in these strains. The findings obtained will serve as crucial baseline data for clinical tracking of *V. vulnificus*, enabling the development of effective measures for the prevention, control, and treatment of *V. vulnificus* infection.

## 2 Materials and methods

### 2.1 *Vibrio vulnificus* collection and identification

*V. vulnificus* isolates from patients were systematically collected at a hospital in Ningbo, Zhejiang Province, China, between 2013 and 2020 (Table 1). To ensure patient privacy, identifying information was erased. The details of these isolates are presented in Table 1. The identification of these isolates was carried out through a combination of conventional microbiological tests and *16S rRNA* gene sequencing. Additionally, confirmation of the identification was carried out using species-specific *vvh* PCR (primer is presented in Supplementary Table S1). All the isolates were preserved in Luria–Berani (LB) broth containing 60% glycerol and stored at −80°C.

**Table 1:** Isolation details of *V. vulnificus* used in this study.

No.	Isolation ID	Year of isolation	Isolation sources	Media used for isolation
1	S1	2018	Blood	Blood Agar
2	S2	2018	Blood	Blood Agar
3	S3	2018	Blood	Blood Agar
4	S4	2019	Blood	Blood Agar
5	S5	2018	Secreta	Blood Agar
6	S6	2021	Secreta	Blood Agar
7	S7	2021	Secreta	Blood Agar
8	S8	2021	Secreta	Blood Agar
9	S9	2020	Blood	Blood Agar
10	S10	2018	Blood	Blood Agar
11	S11	2018	Blood	Blood Agar
12	S12	2020	Secreta	Blood Agar
13	S13	2013	Blood	Blood Agar
14	S14	2014	Blood	Blood Agar
15	S15	2021	Secreta	Blood Agar
16	S16	2018	Secreta	Blood Agar
17	S17	2018	Blood	Blood Agar
18	S18	2018	Blood	Blood Agar
19	S19	2018	Blood	Blood Agar
20	S20	2018	Blood	Blood Agar
21	S21	2020	Secreta	Blood Agar

### 2.2 Analysis of molecular type

*V. vulnificus* strains were grown overnight with shaking (200 rpm) at 37°C in LB medium. The QIAamp DNA Mini Kit (QIAGEN, Shanghai, China) was used for extracting the genomic DNA, following the instructions of the manufacturer. To distinguish between potentially virulent and non-virulent type strains, four loci were targeted, namely, *vcg*, *16S rRNA*, biotype 2, and *Ser* E. The primers used for each locus are presented in Supplementary Table S1. The PCR products were analyzed using gel electrophoresis. Through this method, all strains were characterized by their respective clinical-type *vcg* C or environment-type *vcg* E, as well as their *16S rRNA* type (either *16S rRNA*-A type or -B type), biotype (*bt* 2 or non-*bt* 2), and *ser* E type, as previously reported in the literature (Nilsson et al., 2003; Valiente and Amaro, 2006; Warner and Oliver, 2008).

### 2.3 Antibiotic susceptibility test

The susceptibility of the isolated strains to 15 antibiotics belonging to 6 different classes (Supplementary Table S2) was assessed using the disk diffusion method, as per the recommendations of the Clinical and Laboratory Standard Institute (CLSI) guidelines. *Escherichia coli* ATCC 25922 strains were used as the quality control strains. For the purpose of detailed analysis, isolates exhibiting intermediate susceptibility were considered as susceptible. The multiple antibiotic resistance (MAR) index was determined for each isolate using the formula: MAR index = A/B. In the formula, A represents the number of antibiotics to which the isolate demonstrated resistance, while B represents the total number of antibiotics against which the isolate was tested (Rosengren et al., 2009; Sony et al., 2021). Additionally, the identification of multidrug resistance (MDR) strains was based on their antibiotic-resistant profiles, specifically resistance to at least three or more antibiotics (Sony et al., 2021).

### 2.4 Analysis of susceptibility to serum killing and hemolysis activity

The serum resistance of the isolated strains was assessed using methodologies previously established (Siu et al., 2011). In brief, overnight bacteria cultures were adjusted to a concentration of 1 × 10^6^ CFU/mL. Subsequently, 25 μL of bacteria suspension was mixed with 75 μL of healthy human serum in 96-well plates. The viable bacterial count was determined at 60, 120, and 180 min on LB agar plates. Each strain was tested three times. The results were expressed as a percentage of the initial inoculum, and the survival ability of bacteria in serum was graded from 1 to 6, as previously reported (Podschun et al., 1991; Siu et al., 2011). Specifically, grade 1 represents viable counts of <10% of the inoculum after 1 and 2 h and < 0.1% of the inoculum after 3 h; grade 2 represents viable counts of 10–100% after 1 h and < 10% after 3 h; grade 3 represents viable counts of >100% after 1 h but <100% after 2 and 3 h; grade 4 represents viable counts of >100% after both 1 and 2 h but <100% after 3 h; grade 5 represents viable counts of >100% after 1, 2, and 3 h but decreased during the third hour, and grade 6 represents viable counts of >100% after 1, 2, and 3 h and increased throughout the period. Strains classified as grades 1 or 2 were considered highly sensitive, while those classified as grades 3 and 4 were considered intermediately sensitive and those classified as grades 5 and 6 were considered resistant.

Hemolytic ability was determined using a Columbia CNA blood agar medium (Haibo Biotech Co., LTD, China) containing 20% sheep blood. The cultures were incubated at 37°C for 48 h, following the previous study (Galan-Ladero et al., 2010). The hemolytic ability was determined by calculating the ratio between the diameter of the hemolytic ring and the colony size. A higher ratio indicates a higher level of positive hemolytic activity.

### 2.5 DNA extraction, genome sequencing, and annotation

The genomic DNA was extracted using the QIAamp DNA Mini Kit (QIAGEN, Shanghai, China). Whole-genome sequencing was carried out on an Illumina HiSeq X10 PE150 platform with the exception of the S12 strain, which was sequenced using the Nanopore platform (Biomarker Technologies, Beijing, China). All genomes were annotated using Prokka (Seemann, 2014). The functional classification of coding sequences was conducted using Basic Local Alignment Search Tool (BLAST)^1^ based on the Clusters of Orthologous Genes (COGs) database^2^ and Kyoto Encyclopedia of Genes and Genomes (KEGG).^3^ rRNA and tRNA sequences were identified using RNAmmer (Lagesen et al., 2007) and tRNAscan-SE (Lowe and Chan, 2016), respectively. Virulence genes were analyzed using the virulence factor database (VFDB) 2019 (Liu et al., 2019), and the identification of antibiotic resistance genes was performed using the Comprehensive Antibiotic Resistance Database (CARD) (Alcock et al., 2020). This whole genome shotgun project has been deposited at NCBI under the BioSample accession SAMN41809252-SAMN41809272.

### 2.6 Phylogenetical tree construction

A phylogenetic tree encompassing the dereplicated genomes was constructed based on the concatenation of 120 ubiquitous single-copy marker genes (bac 120 marker set). These genes were identified using HMMER as previously described (Chaumeil et al., 2020). A neighbor-joining phylogenetic tree incorporating 120 genes from all isolates was generated using MEGA 11.0.

### 2.7 Statistical analysis

All statistical analyses were carried out using GraphPad Prism 8.0 (GraphPad Software, Inc., San Diego, CA). Strain comparisons were conducted using either the one-way analysis of variance (ANOVA) with subsequent Tukey’s post-hoc test or Students’s *t*-test. Significant statistical differences were denoted as follows: *p < 0.05, **p < 0.01, ***p < 0.001, and ****p < 0.0001. Error bars are shown in all figures to showcase variations within the data.

## 3 Results

### 3.1 Molecular types of *Vibrio vulnificus* isolates

**Figure 1:**
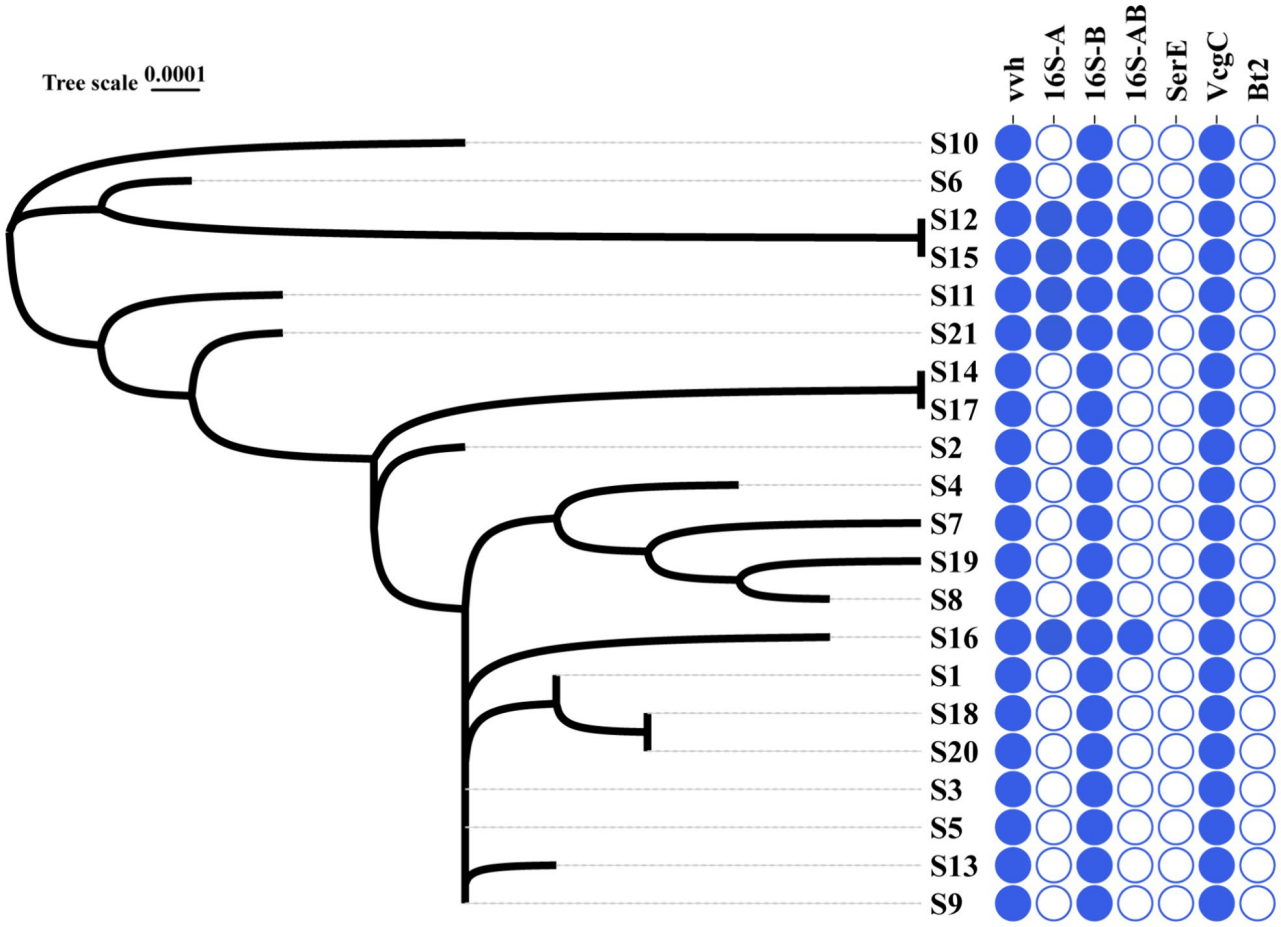
Phylogenic relationship and molecular types of all the *V. vulnificus* isolates. The left graph with S-number designation displays the phylogenic tree of all 21 isolates. The right panel distinguished by different colors illustrates the genotype profiles of all the isolates. Blue represents the presence of genes and white signifies their absence.

*16S* B-*vcg* C type is the most prevalent molecular type of *V. vulnificus* isolates in Ningbo, China. Complete sequencing of the full length of 16S rRNA gene from all 21 *V. vulnificus* isolates obtained from tissues or blood of patients in Ningbo (Zhejiang Province), China, has been conducted (detailed information of isolates is presented Table 1). This species identification has been further confirmed by marker genes of *vvh* (Figure 1 and Supplementary Figure S1). Based on *16S rRNA* gene analysis, serum types (*ser* E), virulence gene-based types (*vcg* C/E), and biotype classification, molecular classification has revealed distinct types of genes among these isolates (Figure 1 and Supplementary Figure S1). All isolates were identified as *vcg* C types, with most of them belonging to *16S* B types. However, five isolates (S12, S15, S11, S21, and S16) were found to carry both *16S* A and B genes. Notably, none of the isolates were of *ser* E and *Bt* 2. Overall, these results suggest that *V. vulnificus* isolated from Ningbo, China, exhibits distinct molecular characteristics.

### 3.2 Antibiotic resistance profiles of *Vibrio vulnificus* isolates

**Figure 2:**
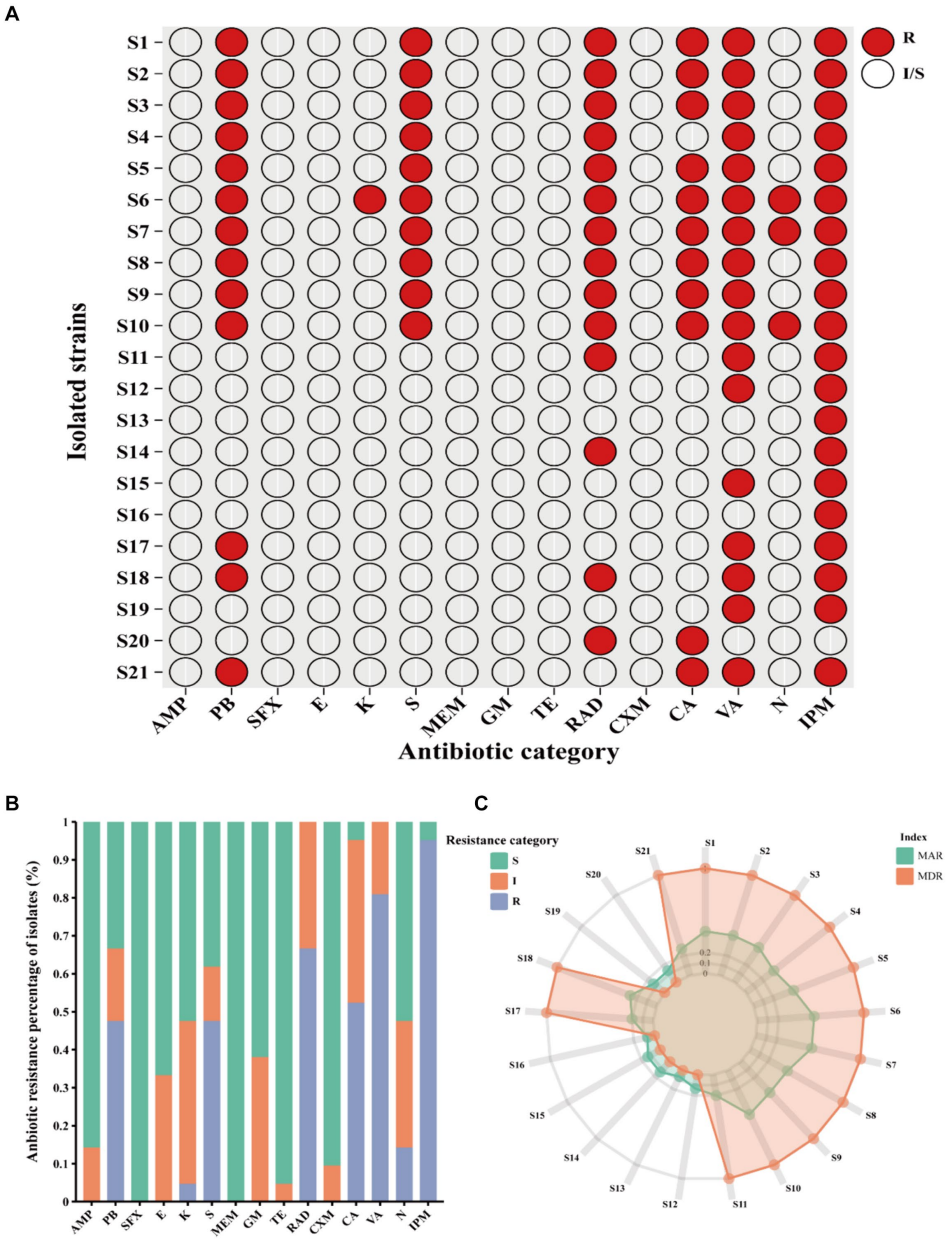
Antibiotic resistance pattern among *V. vulnificus* isolates. **(A)** The antibiotic resistance profiles of 21 isolates. **(B)** The percentage of antibiotic resistance of different isolates. **(C)** MAR and MDR index of all isolates. In the figure, R indicates resistance, I indicates intermediate, and S indicates susceptible.

Clinical *V. vulnificus* isolates exhibited widespread antibiotic resistance. These isolates were found to be commonly resistant to antibiotics, such as polymyxin B (PB), kanamycin (K), streptomycin (S), cefradine (RAD), cefalexin (CA), vancomycin (VA), neomycin (N), and imipenem (IPM) (Figure 2A). Notably, they commonly display resistance to imipenem (IPM, 100%), vancomycin (VA, 80.95%), RAD (66.67%), PB (61.90%), and S (47.62%) (Figure 2B). However, they are mostly sensitive to numerous antibiotics, such as sulfanidazole (SFX, 21 of 21), meropenem (MEM, 21 of 21), tetracycline (TE, 20 of 21), cefuroxime (CXM, 19 of 21), and ampicillin (AMP, 18 of 21) (Figures 2A,B). Notably, different isolates exhibit different antibiotic resistance profiles. Isolates S6 (antibiotic resistance rate of 53.33%), S7 and S10 (antibiotic resistance rate of 46.67%) showed the most widespread antibiotic resistance, aligning closely with a rate of approximately 50% (Figure 2A). Other strains, such as S1, S2, S3, S5, S8 and S9 follow closely behind (antibiotic resistance rate exceeding 40%) (Figure 2A). Conversely, isolates S16 and S13 exhibited sensitivity toward the vast majority of the tested antibiotics being mainly resistant just to IPM. Other notably sensitive strains such as S12, S14, S15, S19, and S20 are resistant to just two tested antibiotics. However, the majority of the isolates were MDR strains with resistance to at least three or more antibiotics observed in 66.7% (Figure 2C). This study shows narratives highlighting significant concerns regarding the usage of antibiotics in the sample population of Ningbo, China. This is further supported by their respective MAR index which ranged from 0.06 to 0.53. Notably, 61.90% of the 21 isolates exhibited a MAR index of more than 0.2, indicating widespread exposure to antibiotics in their environment (Figure 2C). Together, the observed antibiotic resistance profiles of these *V. vulnificus* isolates are of significant concern given their higher prevalence in Ningbo, China.

### 3.3 Serum resistance demonstrated virulence difference of *Vibrio vulnificus* isolates

**Figure 3:**
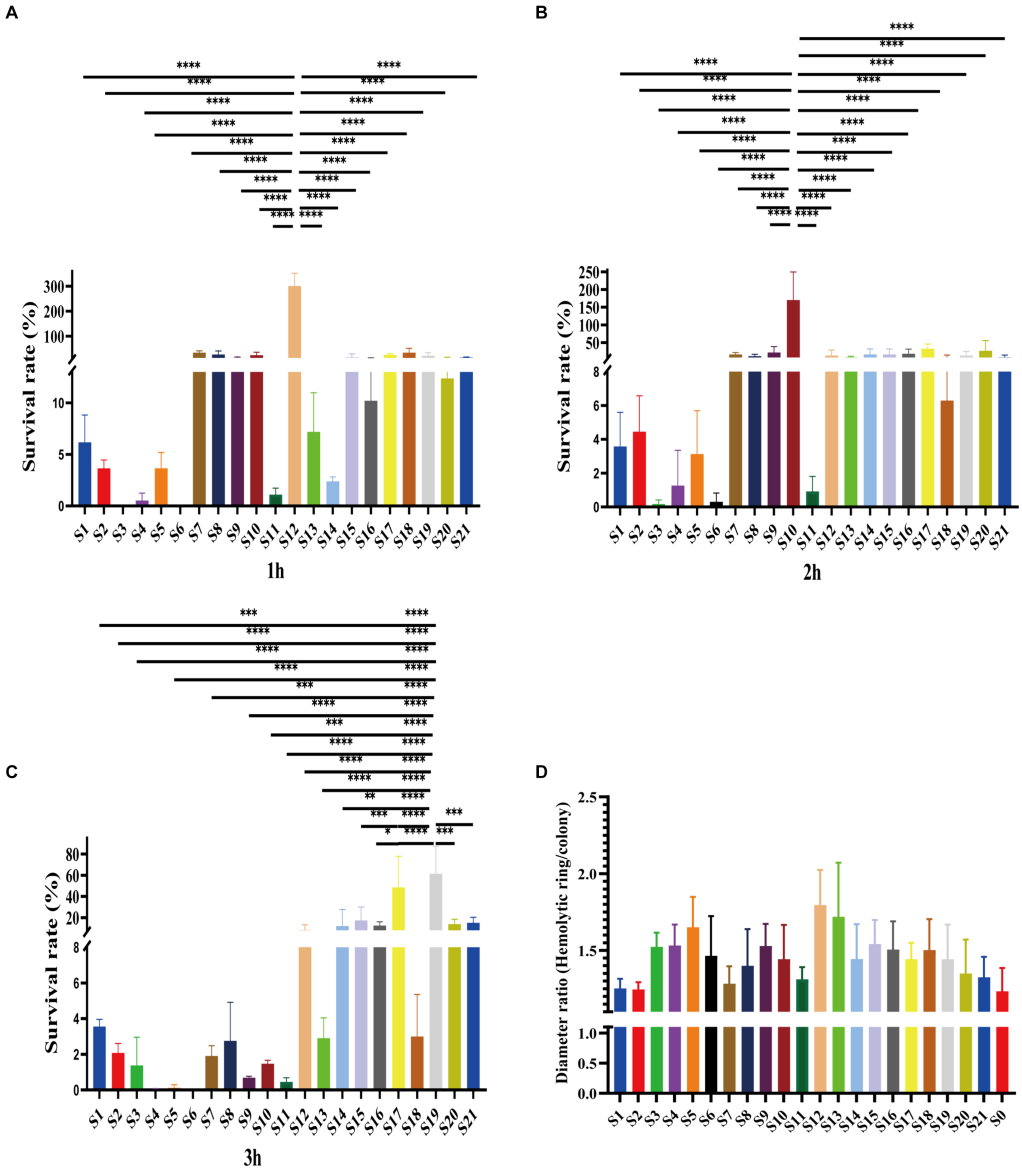
Serum resistance and hemolytic ability of all isolates. **(A–C)**. The survival rates of isolates in serum after 1 h, 2 h, and 3 h. **(D)** Hemolytic ability of isolates indicates by the ratio of hemolytic ring and colony.

Of the 21 clinical isolates, serum resistance tests revealed noteworthy disparities. Consistent with antibiotic resistance difference, the serum resistance of isolates also showed variations. Among them, most were susceptible to serum killing (Figures 3A–C). Specifically, 9 out of 21 isolates belonged to grade 1, while another 11 isolates belonged to grade 2 (Supplementary Figure S2). Especially, one isolate, S12, was observed in the rest of the grade isolates due to its intermediate resistance to serum killing, which was classified as grade 3 (Supplementary Figure S2). This particular strain showed survival rates of 301.16 ± 41.74% after 1 h and 13.77 ± 12.52% and 10.33 ± 3.97% after 3 h (Figures 3A–C). Additionally, this isolate displayed a high hemolytic ring, indicating its higher virulence compared to other strains (Figure 3D). Moreover, although some grade 2 isolates also showed higher survival rates, there were distinct patterns among them. For example, S10 demonstrated significantly higher survival rates after 2 h of co-colonization with serum, and S17 and S19 exhibited higher survival rates after 3 h (Figures 3B,C). These findings suggest varying virulence potential among *V. vulnificus* isolates. The observed difference could have significant implications for understanding the pathogenesis and treatment of infections caused by this bacterium.

### 3.4 Genome properties and annotation

Upon conducting whole genome analysis on all 21 isolates of *V. vulnificus*, they were composed of two genomes. Specifically, the genome size ranged from 4,900,883 bp to 5,296,751 bp with an average of 5,021,634 bp (Supplementary Table S3). The GC content varied from 46.59 to 46.91% with an average of 46.75% (Supplementary Table S3). The total gene sets for all isolates ranged from 4,324 to 4,778 with an average of 4,477 genes (Supplementary Table S3). Of particular note, from the whole nanopore sequencing of S12, we found that this strain has 2 contigs: contig 1 with a length of 3,181,783 bp and contig 2 with a length of 1,868,114 bp (Supplementary Table S3). Additionally, this strain also harbors 4,393 genes, along with 119 rRNA, 34 tRNA, and 65 ncRNA (Supplementary Table S4). In terms of function annotation as per the KEGG, most genes in S12 are involved in metabolism (1534), followed by environmental information processing (367), genetic information processing (233), and cellular processes (180) (Supplementary Figure S3). In summary, the results suggest that different isolates possess distinct genome properties, which could potentially be the main factors contributing to their antibiotic resistance and virulence.

### 3.5 Phylogenic characteristics of the isolates

The phylogenic analysis of 120 ubiquitous single copy genes from 21 isolates revealed genetic relationships among the strains, indicating that they belong to different evolutionary branches (Figure 1). The cladogram showed that S12 is closely clustered with S15, which is then closely clustered with S6 and S10. Notably, this cluster of isolates, except for S6, exhibited higher serum resistance (Figure 1). Other clusters of isolates suggested their close phylogenic relationship: S14 was closely clustered with S17 and S3 and S5 and S9 were closely clustered with S13 (Figure 1). However, the clusters of most isolates did not exhibit identical antibiotic or serum resistance phenotypes, suggesting that related resistance genes may not solely determine the genetic relationship of these isolates. In general, the phylogenetic closeness among all isolates likely reflects the geographical characteristics.

### 3.6 Molecular characteristics of VFs and ARGs

**Figure 4:**
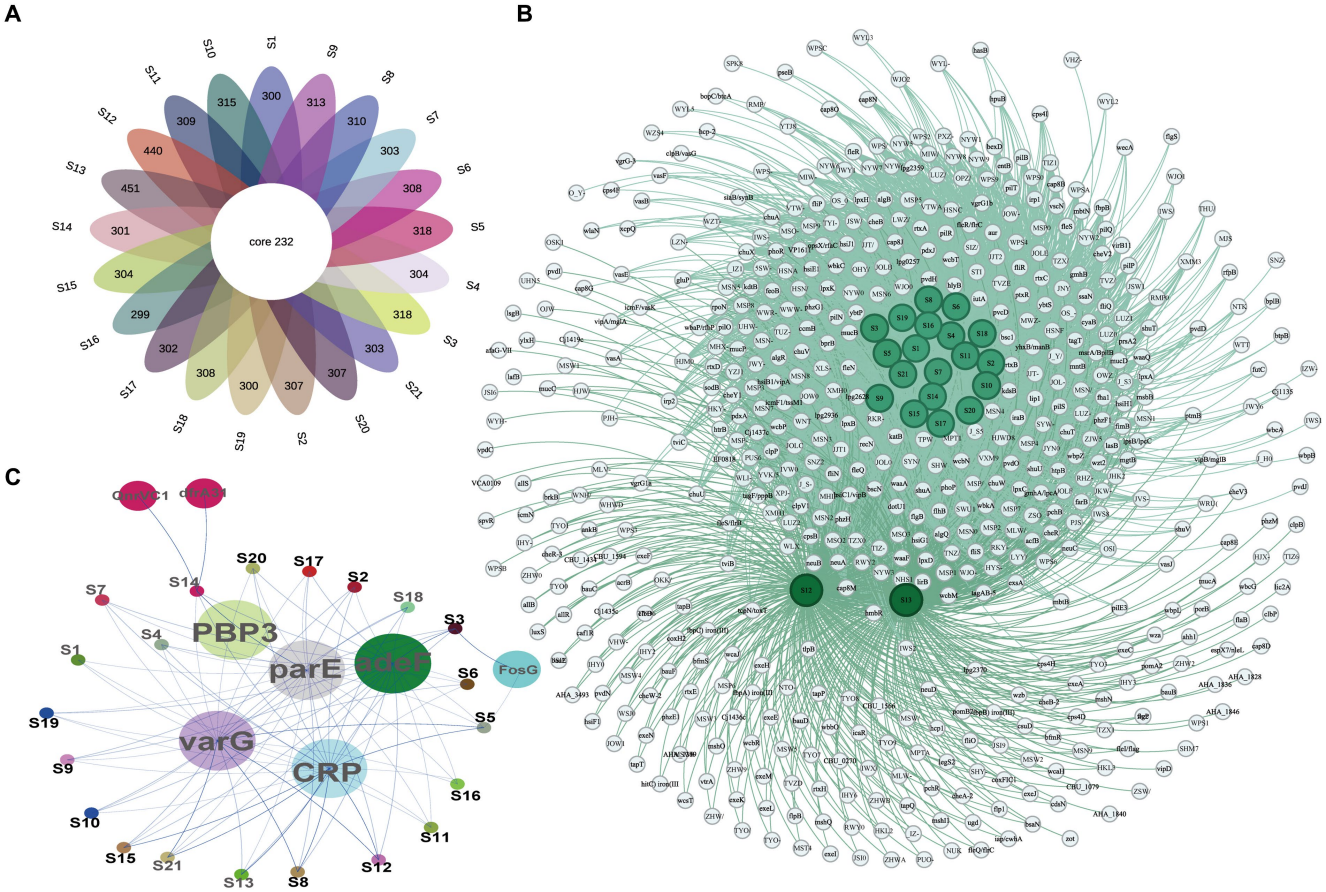
VF and AR gene profiles of all isolates. **(A)** The Venn diagram of VF genes among isolates. **(B)** Co-occurrence network of VF genes among different isolates. **(C)** Co-occurrence network of ARGs among different isolates.

The ARGs and VFs of all isolates were annotated. Our analysis revealed that different isolates possess distinct ARGs and VFs. As to VFs, the 21 isolates examined had VFs ranging from 531 to 683, with an average of 558.81 (Supplementary Table S5). Among all these strains, isolates S12 and S13 exhibited the highest number of VFs (683 and 672, respectively) (Supplementary Table S5), while isolates S16 and S19 had the lowest number of VFs (531 and 532, respectively) (Supplementary Table S5). This suggests different exhibit varying degrees of VFs which may determine their virulence. Moreover, these isolates shared 232 genes of VFs, including adherence (fimbriae and pili), iron uptake, siderophore, anti-phagocytosis, endotoxin (lipopolysaccharide), secretion systems (type II, type III, type IV, and type VII), chemotaxis, and motility (flagella proteins), enzyme and toxin (hemolysin, cytolysin, MARTX, RTX, and metalloprotease) (Figures 4A,B and Table 2). Specifically, gene-encoding enzymes and toxin factors such as cytolysin *cyl*A, hemolysin *hlyD*, *hlyB,* and *hlyA* (*vvh*), MARTX gene clusters *rtx ABCD,* and metalloprotease *strc*E were prevalent in all these strains (Figures 4A,B and Table 2). Other common VFs, including CPS genes such as *cps*AB, *kps*F, *cysC*, *cj*1437, *cap8J*, *bsc1*, *wzt2*, and *wcbTPN,* were also prevalent (Figures 4A,B and Table 2). The widespread prevalence of these co-shared VFs suggests strong infection potential toward the host, highlighting the higher toxic molecular characteristics of clinical isolates in Ningbo, China. Additionally, the isolates also displayed varying patterns of ARGs. Common ARGs in *V. vulnificus* were also detected in all isolates such as *PBP*3, *parE*, *adeF*, *varG,* and *CRP,* which mainly confer resistance to antibiotics, such as beta-lactams, fluoroquinolones, and carbapenem (Figure 4C). Specifically, S3 and S5 shared the *FosG*, conferring resistance to fosfomycin, while S14 possessed *QnrVC1* and *dfrA31* related to resistance to quinolone and diaminopyrimidine antibiotics (Figure 4C). These findings explain the observed antibiotic resistance profiles. For example, *varG* may coordinate with the higher resistance observed in isolates toward vancomycin (80.95%), while other ARGs such as *CRP* are likely to explain the higher resistance to imipenem (carbapenem, 100%). However, there is still a discrepancy between the presence of ARGs and the corresponding antibiotic resistance phenotype. For instance, although *adeF* is present in all isolates, they do not exhibit increased resistance to tetracycline. Taken together, these results suggest that the presence of distinct ARGs and VFs in different isolates determines their respective antibiotic resistance and virulence characteristics.

**Table 2:** Major co-shared VF gene profiles of all isolates based on VFDB.

Virulence class	Virulence factor and genes
Adherence	Accessory colonization factor (*acfB*), Campylobacter adhesion to fibronectin (*cadF*), Heat shock protein (*htpB*), Immunogenic lipoprotein A (*llpA*), InternalinJ (*inlJ*), Laminin-binding protein (*lmb*), Multivalent adhesion molecule 7 (*VP1611*), Cell-binding factor (*pebA*), Type I fimbriae (*fimE*, *fimB*), Type IV pili (*pilS*, *pilR*, *pilO*, *pilN*, *pilH, chpD*)
Iron uptake and siderophore	Heme uptake (*chuX*, *chuW*, *chuV*, *chuT*, *shuU*, *shuA*), Enterobactin (*fepC*, *entA*, *entF*, *entE*, *entD*), methyltransferase (*iraB*), Legiobactin (*lbtC*), Pyochelin (*pchD*, *pchB*, *pchA*, *fptA*), Yersiniabactin (*ybtS*, *ybtP*, *ybtE*)
Antiphagocytosis	Mucoid exopolysaccharide (*mucB*, *algZ*, *algU*, *algR*, *algQ*, *algB*, *algC*, *mucP*, *mucD*, *mucB*, *algW*), Capsule (*kpsF*, *cysC*, *cpsB*, *cpsA*, *Cj1437c*, *cap8J*, *bsc1*), Capsule I (*wzt2*, *wcbT*, *wcbP*, *wcbN*), Hyaluronic acid capsule (*hasC*)
Endotoxin	Lipooligosaccharide (*yhxB*/*manB*, *waaQ*, *rfaE*, *rfaD*, *orfM*, *opsX*/*rfaC*, *msbB*, *IsgC*, *lpxK*, *lpxH*, *lpxC*, *lpxB*, *lpxA*, *lgtF*, *kdsB*, *kdsA*, *kdkA*, *htrB*, *gmhB*, *gmhA*/*lpcA*, *galE*), Lipopolysaccharide (*wbpZ*, *wbkC*, *wbkA*, *waaF*, *waaA*, pgm, *lpsB*/*lpcC*, *kdtB*, *fabZ*, *bplA*, *acpXL*)
Secretion system	Type III secretion system (*bprB*, *bscN*, *ssaN*), Type IV secretion system (*lpnE*, *lpg2936*, *lpg2628*, *lpg0257*, *lirB*), Type II secretion system (*gspG*, *gspD*), Type VII secretion system (*essC*)
Chemotaxis and motility	Flagella (*filC, flhG, flgL, flgK, cheY1, cheR, cheB, filR, filQ, filP, filN, filM, filL, filK, filG, filF, filE, filA,filS, flhF, flhB, flhA, flqJ, flgI, flgH, flgG, flgF, flgE, flgD, flgC, flgB, flgA, fleR/flrC, fleN, fleQ,MotY*), Chemotaxis (*cheZ, cheY, cheV, cheA, cheD, cheW*)
Enzyme and toxin	Aureolysin (*aur*), Cytolysin (*cylA*), Hemolysin (*hlyD*, *hlyB*, *hlyA*), Elastase (*lasB*), MARTX (*rtxA*), RTX toxin (*rtxD*, *rtxC*, *rtxB*), Macrophage infectivity potentiator (*mip*), Beta-hemolysin/cytolysin (*cyll*, *cylG*), signal peptidase II (*lspA*), Metalloprotease (*stcE*)
Other	Adenosine synthase A (*adsA*), arylsulfatase (*AslA*), cyclic beta 1–2 glucan synthetase (*cgs*), cyclic di-GMP phosphodiesterase (*cdpA*), Cytochrome c maturation locus (*ccmF*, *ccmE*, *ccmB*, *ccmA*), Stress protein (*clpC*, *clpP*, *katA*, *katB*, *recN*, *sodB*, *sodCI*),Fatty acid efflux system protein (*farB*, *farA*), Neutrophil activating protein (*napA*), Hexose phosphate transport protein (*hpt*), Hyaluronic acid capsule (*hasC*), Mg^2+^ transport protein (*mgtC*, *mgtB*),Multiple transferable resistance system (*mtrD*, *mtrC*), Phenazines biosynthesis (*phzH*, *phzG1*, *phzF1*), Regulation (*phoP*, *phoR*), Post translocation chaperone (*prsA2*), (*pdxJ*, *pdxA*, *rpoN*, *eptC*), pyoverdine (*pvdO*, *pvdH*,*ptxR*, *pvcD*), Intracellular survival (*ricA*)

## 4 Discussion

*V. vulnificus* infection is rare but potentially fatal. Therefore, it is important to investigate antibiotic resistance and virulence traits to effectively control and prevent its spread. In this study, we retrospectively examined the molecular characteristics of 21 clinical isolates in Ningbo, China. Our findings indicate that *V. vulnificus* strains circulating in Ningbo, Zhejiang, China, exhibit increased antibiotic resistance and virulence. Notably, instead of their distinct types and phylogenetic relationship, these isolates shared a number of ARGs and VF genes, indicating their geographical coherence and the potential for cross-transmission.

There are three biotypes in *V. vulnificus* strains based on their biochemical characteristics. Biotype 1 is predominantly responsible for human infection and widely distributed worldwide. Biotype 2 primarily affects eel pathogenicity. Although biotype 3 strains have been reported to cause human infections, they are currently limited to Israel (Jones and Oliver, 2009). Through molecular typing systems, we confirmed that all 21 *V. vulnificus* isolates from Ningbo, China, were not of biotype 2. Biotype 2 is characterized by a single type of LPS, which resulted in its designation as serogroup E (Biosca et al., 1996). Consequently, these 21 isolates were also detected to be non-ser E type. In correlation with the restricted location of biotype 3 in Israel, we speculate that all these strains may belong to biotype 1, which were isolated from the infected individuals. However, it needs further confirmation. In addition to the biotype classification, *V. vulnificus* is usually categorized based on their environmental or clinical sources and genotypes, which suggest different virulence potentials. Based on this method, we observed that all isolates belonged to *vcg* C type, and the majority (16 of 21) of them were classified as *16S rRNA* B types, which have previously been recognized as potentially more virulent in clinical isolates (Warner and Oliver, 2008). This type of *V. vulnificus* is frequently detected in seafood, particularly in Hangzhou and Zhoushan, located in Hangzhou Bay near Ningbo (Pan et al., 2013). The positive proportion is even higher, reaching 86.3% in oysters in Zhoushan, which is located close to Ningbo City and is a significant seafood supplier to the region (Pan et al., 2013). These findings suggest a potential connection between clinical *V. vulnificus* isolates in Ningbo and seafood sourced from the region. Notably, studies have linked the local population’s preference for raw seafood consumption with *V. vulnificus* infections (Zheng and Sheng, 2016; Wang et al., 2023). Given the high seafood demand and ongoing climate deterioration, environmental *V. vulnificus* may serve as a reservoir with potential virulence, posing a risk for disease outbreaks. Therefore, environmental prevention and control measures become crucial in mitigating *V. vulnificus* outbreak.

*Vibrio* species are usually sensitive to most antibiotics commonly used in veterinary and human medicine. Nevertheless, several studies have reported the emergence and evolution of multiple antibiotic resistance profiles among these strains due to the misuse and excessive use of antibiotics in humans, agriculture, and mariculture (Elmahdi et al., 2016). In line with previous studies, we have observed an increase in antibiotic resistance among clinical *V. vulnificus* (Pan et al., 2013; Elmahdi et al., 2016). The high MDR rate of 66.7% among the 21 isolates confirmed these findings, which are also higher compared with the rates reported in the USA (Baker-Austin et al., 2009). Notably, most isolates have demonstrated resistance to more than three antibiotics. Importantly, there is a concerning persistence of high resistance among these strains to cephalosporins, such as cefalexin and cefradine, which is a significant concern for clinicians treating *V. vulnificus* infections (Pan et al., 2013). Moreover, our study has identified emerging resistance among these isolates to imipenem and vancomycin, which were less frequently reported previously, indicating an alteration in the antibiotic resistance of *V. vulnificus* (Sony et al., 2021). This is supported by the MAR index, with over 60% of isolates exhibiting a MAR index of >0.2, suggesting that they originated from the sources of high-antibiotic resistance risks. This high MAR index for *V. vulnificus* has also been observed by Sony et al. (2021), who reported a high rate of (75%) antibiotic resistance among *Vibrio* isolates in Malaysia (Mohamad et al., 2019). The higher MAR suggests that environmental antibiotic resistance may play an important role in the antibiotic resistance pattern of clinically infected *V. vulnificus*. This aligns with the previous reports of high antibiotic resistance in *V. vulnificus* in seafood and related infection patients in Hangzhou Bay areas (Pan et al., 2013; Zheng and Sheng, 2016; Weng et al., 2021; Wang et al., 2023). However, it is noteworthy that most of the isolates are fully susceptible to ampicillin, sulfafurazole, erythromycin, meropenem, gentamicin, tetracycline, and cefuroxime. This susceptibility pattern to antibiotics has been substantiated by multiple previous studies, which are conducted not just in China but also in other countries such as Iran and Singapore (Raissy et al., 2012; Pan et al., 2013; Ng et al., 2018). These varying resistant antibiotics suggest that treatment should not only be based on a medication guide but also tailored to individual antibiotics through susceptibility testing. Additionally, to better understand the relationship between antibiotic resistance profiles, we investigated the genetic basis of ARGs among the clinical isolates. Consistent with the results of antibiotic resistance testing, we identified prevalent ARGs, such as *PBP3*, *parE*, *adeF*, *varG,* and *CRP*. These genes may confer resistance to cephalosporin, fluoroquinolone, macrolide, and vancomycin antibiotics, respectively. This provides a partial explanation for the observed antibiotic resistance phenotype. However, there is still an unexplained discrepancy between ARGs and antibiotic resistance that requires further investigation to thoroughly analyze genetic variations and exchanges. Moreover, the association between *vcg* and *16S rRNA* types and antibiotic resistance requires further exploration and confirmation.

The virulence of isolates varies significantly due to differences in their VFs. Based on our analysis, all 21 isolates were found to possess a range of VF genes, including important key ones, such as CPS, iron acquisition, flagella and motility, hemolysin/cytolysin, metalloprotease, and RTX toxin. These factors may significantly contribute to their high virulence, which has been found prevalent in *vcg* C-*16S rRNA* B type, as identified in our study (Warner and Oliver, 2008). However, as of now, no specific VF has been identified to distinctly characterize the virulent strains (Ng et al., 2018). The only virulence factor that has been established as essential for *V. vulnificus* infection is CPS (Baker-Austin et al., 2018). Several studies have demonstrated that CPS enables *V. vulnificus* to resist serum and phagocytosis by macrophages (Johnson et al., 1984; Strom and Paranjpye, 2000; Baker-Austin et al., 2018). Specifically, there is a positive correlation between CPS and virulence (Strom and Paranjpye, 2000). The presence of CPS-encoding genes, such as *cpsA/B*, *kpsF*, *cysC*, *bac1*, *cap8J*, *wzt2*, and *wcbT/P/N*, in all isolates suggests a higher virulence potential of these strains. Moreover, once ingested into the body, other VFs such as flagella, pili, and iron acquisition systems likely contribute to bacterial attachment, colonization, and propagation (Simpson and Oliver, 1983; Strom and Lory, 1993; Ashrafian, 2003; Kim and Rhee, 2003; Lee et al., 2004; Jones and Oliver, 2009). Therefore, we found the prevalence of flagella-coding VF genes such as *filC/R/Q/P/N/M/L/K/G/F/E/A/S*, *flhG/A/F/B* and f*lg L/K//I/H/G/F/E/D/C/B/A,* and *cheR/B/Y1*, pili-associated VF genes such as *pilS/R/O/N/H* and *chpD*, heme uptake-associated VF genes such as *chuX/W/V/T* and *shuU/A* in all infected *V. vulnificus* isolates. Additionally, surviving *V. vulnificus* produce toxins to elicit host response. The most commonly known toxin including hemolysin and cytolysin VF genes were frequently observed in our study such as *cylA*, *hylD*/*B/A*, and *cylL/G* (Strom and Paranjpye, 2000). In line with the presence of these genes, we detected the hemolysin ring of all the strains. The results also demonstrated hemolytic ability among isolates, suggesting their potential different virulence. Furthermore, we identified other toxin-coding genes such as enterobactin *fepC*, *entA/F/E/D*, ligiobactin *lbtC*, yersiniabactin *ybtS*, *ybtP*, *ybtE,* and metalloprotease *stcE*. Importantly, the recently identified and extensively studied VF, the RTX family of toxins, was universally present in these isolates (Strom and Paranjpye, 2000). The RTX operon consists of four genes: *rtxA* encodes the toxin, *rtxC* encodes the essential acylase of *rtxA, rtxB* encodes ATP-binding cassette transporter for *rtxA*, and *rtxD* encodes a gene with unknown function in transport. Studies have proven that *rtxA* promotes *V. vulnificus* to rapid growth and epithelial tissue necrosis during intestinal infection (Blanke et al., 2012; Lee et al., 2020), which is essential for bacterial dissemination from the intestine (Gavin et al., 2017). The prevalence of *rtx* operon in all isolates suggests that it may be a critical virulence factor for infection occurrence and could serve as one of the markers of virulent *V. vulnificus*. Otherwise, we also found type II, III, IV, and VI secretion systems prevalent in all isolates. These systems mediate toxin release and bacterial translocation into host cells, which play an important role in infection that warrants further investigation. Collectively, the 21 clinical *V. vulnificus* isolates possessed an abundance of VF genes, which may be the main reason for their infection occurrence. For instance, the most serum resistant strain S12 possessed one of the highest numbers of VF genes. However, the causal relation between VF genes and infection needs further analysis, as strains belonging to different *vcg*-*16 s rRNA* types may exhibit varying virulence. This suggests that these isolates may cause infections through different mechanisms. Future comparisons between our clinical isolates and environment strains or other sources of *V. vulnificus* will provide a better understanding of characteristics of virulence genes.

In summary, our study analyzed the antibiotic resistance and virulence of 21 clinical *V. vulnificus* isolates from Ningbo, China. We explored the relationship between these phenotypes and associated ARGs and VF genes, thereby illustrating the characteristics of these genes among different strains. This study provides fundamental insights for the prevention, control, and treatment of clinical *V. vulnificus*. Based on our results, several strategies can be recommended for the prevention of *V. vulnificus*. First, it is crucial to strengthen surveillance efforts, encompassing both clinical and environmental samples to monitor the presence of *V. vulnificus* strains, as well as their virulence and antibiotic resistance profiles. This comprehensive surveillance will facilitate early detection and timely interventions. Moreover, it is essential to study the ecological dynamics of *V. vulnificus* in relation to changing environmental conditions, particularly under the influence of climate change. Anticipating shifts in transmission patterns and implementing targeted interventions in vulnerable regions can help address the potential impact of climate change on the geographical spread of *V. vulnificus*. Additionally, the development of rapid and accurate strain-typing methods for *V. vulnificus* strains can enable prompt diagnosis and appropriate treatment, thereby improving patient outcomes and reducing the spread of the pathogen. Furthermore, public awareness campaigns should be implemented to educate individuals about the importance of prevention of environmental pollution such as avoiding the misuse of antibiotics, practicing proper seafood consumption, and handling and taking precautions to avoid contact with seawater, especially in the presence of wounds. Such measures can significantly reduce the risk of *V. vulnificus* infections. Continued research and multidisciplinary efforts are necessary to further advance our understanding of this pathogen and improve public health outcomes.

## Data availability statement

The datasets presented in this study can be found in online repositories. The names of the repository/repositories and accession number(s) can be found in the article/Supplementary material.

## Ethics statement

The studies involving humans were approved by The Ethics Committee of Ningbo No.2 Hospital. The studies were conducted in accordance with the local legislation and institutional requirements. Written informed consent for participation was not required from the participants or the participants' legal guardians/next of kin in accordance with the national legislation and institutional requirements.

## Author contributions

XX: Conceptualization, Methodology, Writing – review & editing. SL: Investigation, Methodology, Software, Writing – review & editing. XinL: Investigation, Methodology, Writing – review & editing. WH: Methodology, Writing – review & editing. XiL: Investigation, Software, Writing – review & editing. LL: Methodology, Software, Writing – review & editing. HL: Writing – original draft, Writing – review & editing, Conceptualization, Funding acquisition, Methodology.

## Footnotes

^1^https://blast.ncbi.nlm.nih.gov/Blast.cgi

^2^http://www.ncbi.nlm.nih.gov/COG/

^3^https://www.kegg.jp/
