# Supplementary material for: Antibiotic resistance and virulence characteristics of Vibrio vulnificus isolated from Ningbo, China
Source: Front Microbiol. 2024 Aug 5;15:1459466. doi: 10.3389/fmicb.2024.1459466 (PMC11330838; doi:10.3389/fmicb.2024.1459466)
Supplement: Supplementary file 1 [file Data_Sheet_1.docx]

**Supplementary information for**

**Antibiotic Resistance and Virulence Characteristics of Vibrio Vulnificus Isolated from Ningbo, China**

Xiaomin Xu^1†^, Shanyan Liang^1†^, Xin Li^2^, Wenjin Hu^2^, Xi Li^2^, Liusheng Lei^2^, Huai Lin^2,3*^

^1^Department of Hospital Infection Management, Ningbo No. 2 Hospital, Ningbo, China

^2^State Key Laboratory of Pollution Control and Resource Reuse, School of the Environment, Nanjing University, Nanjing, China

^3^Shenzhen Research Institute of Nanjing University, Shen Zhen, China

^†^These authors contributed equally to this work

*Corresponding Author: [linhuai@nju.edu.cn](mailto:linhuai@nju.edu.cn)

**Summary Information**

**5 Tables**

**3 Figures**


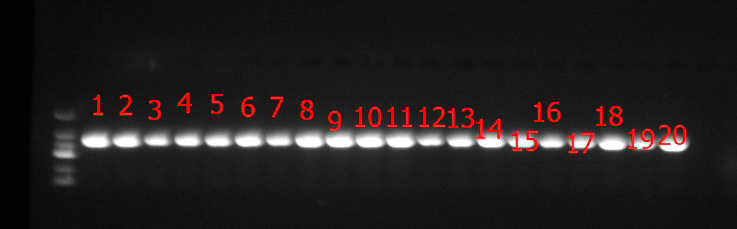

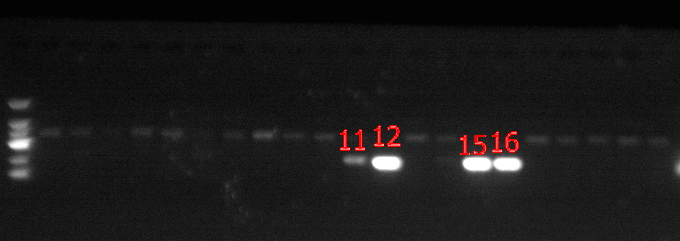


**a.**

**b.**


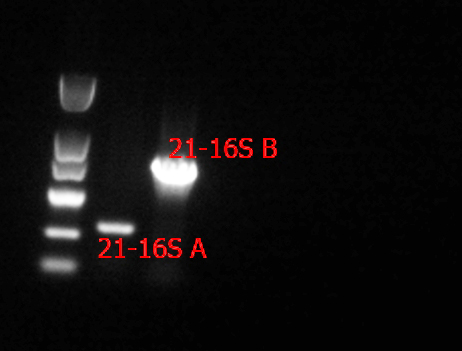


**c.**


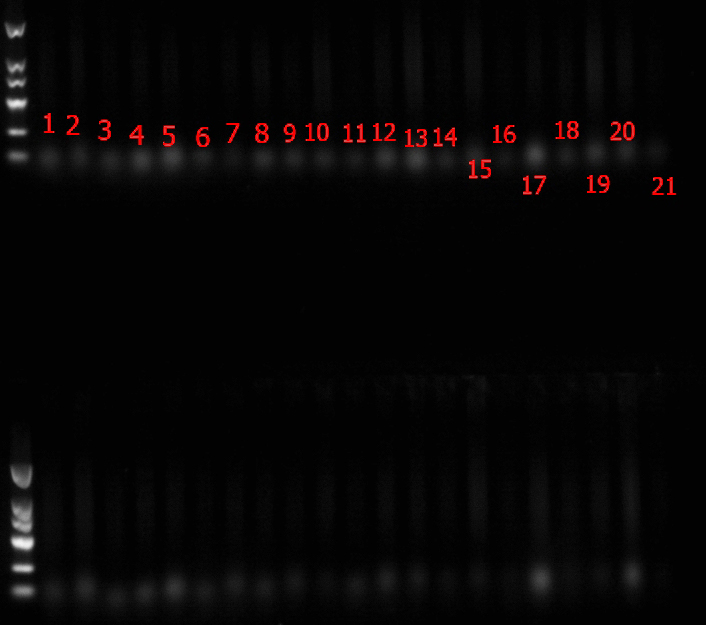


**d.**


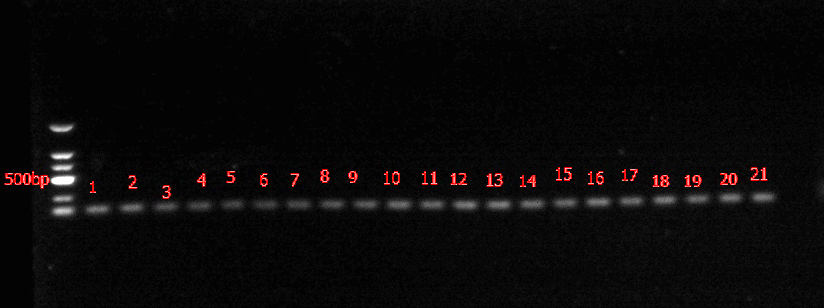


**e.**


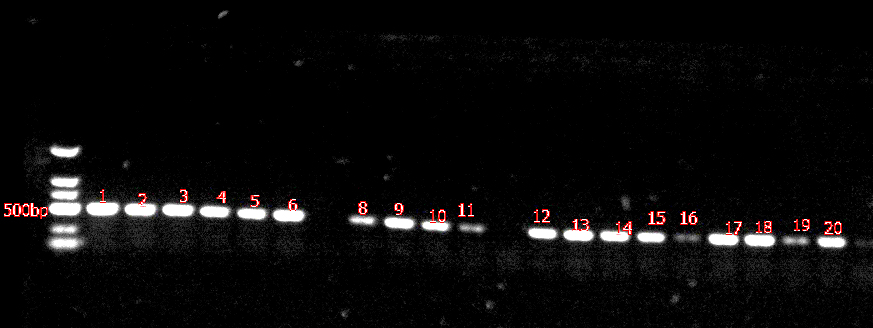

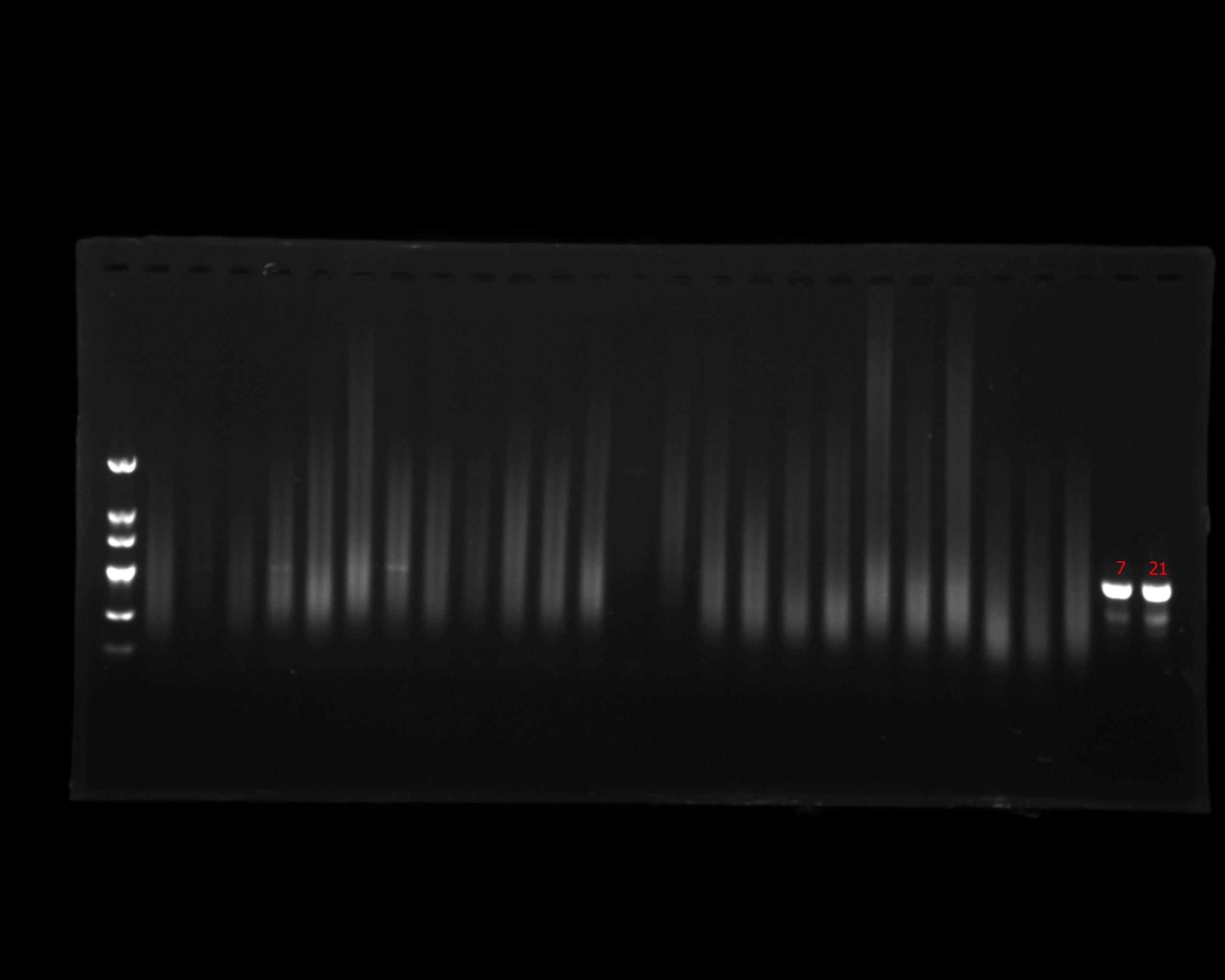


**f.**

**g.**

**FIGURE. S1** Agarose gel eletrophoresis of the PCR products of marker genes.

a and b. Electrophoretic profiles of the *vvh* genes (519bp) of all isolates. c and d. 1*6S rRNA* B gene products (839bp) of all isolates. e. *16S rRNA* A (285bp) gene products of all isolates. f. *ser*E and *bt*2 gene products of all isolates. g. *vcg* C gene products of all isolates. Marker used in this study are DL2000, the strip of it from the bottom up is 100bp, 250bp, 500bp, 750bp, 1000bp and 2000bp.

**
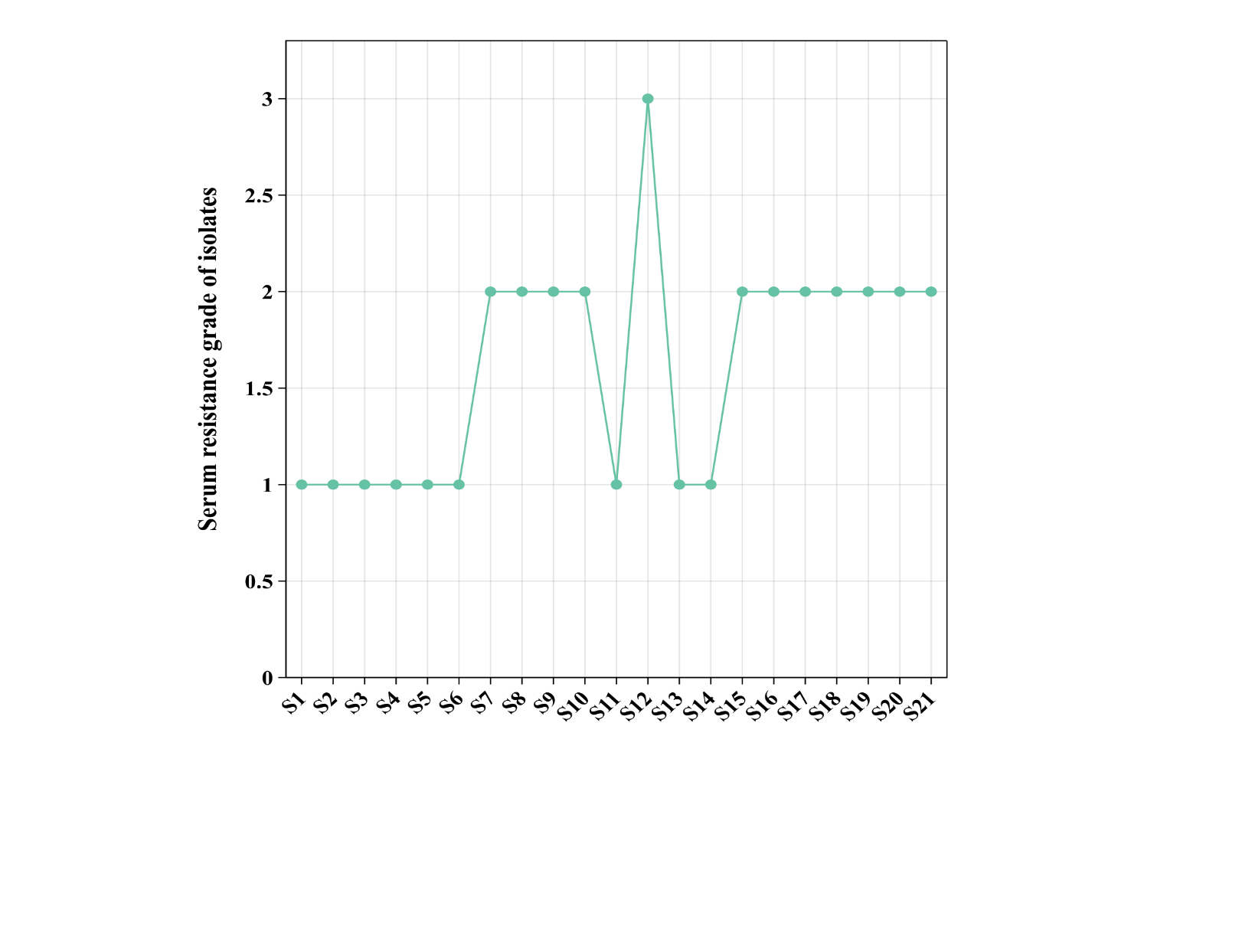
**

**FIGURE S2.** Serum resistance grade of all isolates.


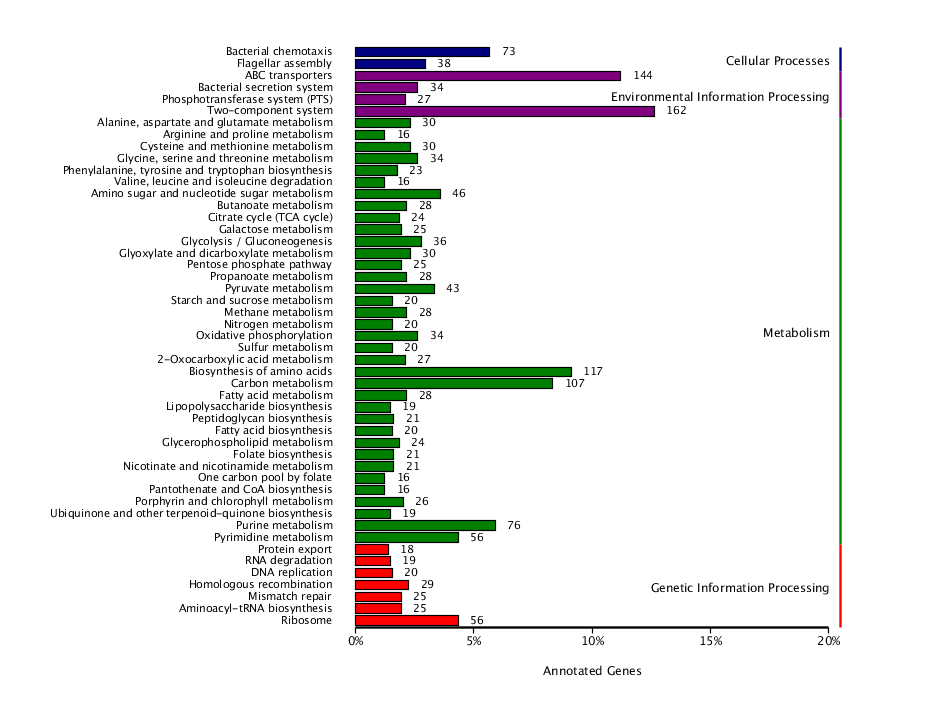


**FIGURE S3.** Top 50 functional classification of the protein coding sequences in S12 isolate based on KEGG categories.

**TABLE S1.** Primers of molecular type identification genes used in this study.

| **ARGs** | **Primers** | | **Amplification length** | **Annealing temperature (℃)** | **Ref** |
| --- | --- | --- | --- | --- | --- |
|  | **Forward** | **Reverse** |  |  |  |
| *vvh* | CCGCGGTACAGGTTGGCGCA | CGCCACCCACTTTCGGGCC | 519 bp | 62 | [1, 2] |
| *vcgC* | AGCTGCCGATAGCGATCT | TGAGCTAACGCGAGTAGTGAG | 97 bp | 55 | [3] |
| vcgE | CTCAGAAAGGCTCAATTGAC | GATTAACGCTGTAAGGCCG | 199 bp | 55 | [3] |
| 16S rRNA A | CATGATAGCTTCGGCTCAA | CACTACCACCTTCCTCACGAC | 285 bp | 58 | [4] |
| 16S rRNA B | GCCTACGGGCCAAAGAGG | CCTGCGTCTCCGCTGGCT | 839 bp | 58 | [4] |
| S*erE* | TGTTGTTCTTGCCCACTCTC | CGCGCTTAGATTTCTCTCACC | 665 bp | 64 | [2, 5] |
| *Bt2* | AGAGATGGAAGAAACAGGCG | GGACAGATATAAGGGCAAATGG | 344 bp | 64 | [5] |

**TABLE S2.** Antibiotic disc used in this study

| **No.** | **Code** | **Antibiotics** | **Subclass** | **Major class of antibiotics** |
| --- | --- | --- | --- | --- |
| 1 | AMP^10^ | Ampicillin | Penicillins | β-lactams |
| 2 | RAD^30^ | Cefradine | First generation cephalosporins |  |
| 3 | CA^30^ | Cefalexin | First generation cephalosporins |  |
| 4 | CXM^30^ | Cefuroxime | Second generation cephalosporins |  |
| 5 | MEM^10^ | Meropenem | Carbapenems | Carbapenems |
| 6 | IPM^10^ | Imipenem |  |  |
| 7 | K^30^ | Kanamycin | Aminoglycosides | Aminoglycoside |
| 8 | S^10^ | Streptomycin |  |  |
| 9 | GM^10^ | Gentamicin |  |  |
| 10 | N^30^ | Neomycin |  |  |
| 11 | TE^30^ | Tetracycline | Tetracyclines | Tetracycline |
| 12 | E^15^ | Erythromycin | Macrolide | Macrolide |
| 13 | PB^300IU^ | PolymyxinB | Polypetides | Polypetide |
| 14 | VA^30^ | Vancomycin | Vancomycin |  |
| 15 | SFX^300^ | Sulfafurazole | Sulfonamide | Sfonamide |

**TABLE S3.** Summary of whole-genome sequencing for 21 V. vulnificus isolates

| **Sample** | **Scaffold Length(bp)** | **Scaffold Number** | **Scaffold N50(bp)** | **Scaffold N90(bp)** | **Contig Length(bp)** | **Contig Number** | **Contig N50(bp)** | **Contig N90(bp)** | **GC Content (%)** | **Geneset Number** |
| --- | --- | --- | --- | --- | --- | --- | --- | --- | --- | --- |
| S1 | 4,900,883 | 89 | 276,452 | 117,129 | 4,900,883 | 89 | 276,452 | 117,129 | 46.91 | 4,324 |
| S2 | 4,981,399 | 125 | 421,301 | 80,442 | 4,981,399 | 125 | 421,301 | 80,442 | 46.73 | 4,409 |
| S3 | 4,998,361 | 133 | 362,736 | 101,609 | 4,998,361 | 133 | 362,736 | 101,609 | 46.79 | 4,445 |
| S4 | 4,966,792 | 147 | 426,466 | 81,058 | 4,966,792 | 147 | 426,466 | 81,058 | 46.81 | 4,454 |
| S5 | 4,997,208 | 131 | 402,664 | 101,617 | 4,997,208 | 131 | 402,664 | 101,617 | 46.79 | 4,444 |
| S6 | 5,173,588 | 120 | 422,631 | 73,346 | 5,173,588 | 120 | 422,631 | 73,346 | 46.66 | 4,637 |
| S7 | 5,038,925 | 94 | 201,007 | 64,933 | 5,038,925 | 94 | 201,007 | 64,933 | 46.8 | 4,454 |
| S8 | 5,296,751 | 136 | 262,511 | 66,932 | 5,296,751 | 136 | 262,511 | 66,932 | 46.6 | 4,778 |
| S9 | 4,964,226 | 83 | 351,686 | 155,740 | 4,964,226 | 83 | 351,686 | 155,740 | 46.75 | 4,358 |
| S10 | 5,258,987 | 142 | 380,700 | 72,908 | 5,257,865 | 145 | 378,193 | 72,908 | 46.63 | 4,708 |
| S11 | 4,950,704 | 65 | 843,421 | 117,087 | 4,950,671 | 66 | 573,367 | 117,087 | 46.83 | 4,392 |
| **S12** | **5,049,897** | **2** | **3,181,783** | **1,868,114** | **5,049,897** | **2** | **3,181,783** | **1,868,114** | **46.77** | **4,392** |
| S13 | 5,012,527 | 34 | 631,138 | 140,322 | 5,012,527 | 34 | 631,138 | 140,322 | 46.67 | 4,391 |
| S14 | 4,940,137 | 130 | 315,528 | 80,372 | 4,940,137 | 130 | 315,528 | 80,372 | 46.84 | 4,370 |
| S15 | 4,974,922 | 103 | 264,715 | 80,760 | 4,974,922 | 103 | 264,715 | 80,760 | 46.69 | 4,404 |
| S16 | 4,930,525 | 88 | 275,941 | 79,520 | 4,930,525 | 88 | 275,941 | 79,520 | 46.86 | 4,357 |
| S17 | 4,986,380 | 108 | 360,230 | 101,388 | 4,986,380 | 108 | 360,230 | 101,388 | 46.82 | 4,453 |
| S18 | 4,971,167 | 105 | 418,439 | 71,434 | 4,971,134 | 106 | 418,439 | 71,434 | 46.78 | 4,420 |
| S19 | 4,937,829 | 113 | 424,526 | 123,548 | 4,937,829 | 113 | 424,526 | 123,548 | 46.71 | 4,391 |
| S20 | 4,937,451 | 90 | 398,228 | 94,195 | 4,937,451 | 90 | 398,228 | 94,195 | 46.82 | 4,378 |
| S21 | 5,185,651 | 157 | 424,569 | 81,079 | 5,185,136 | 159 | 424,569 | 81,079 | 46.59 | 4,687 |

**TABLE S4.** Summary of whole-genome sequencing of S12 isolates

| Property | S12 | | |
| --- | --- | --- | --- |
| RNA | rRNA  34 | tRNA  119 | ncRNA  65 |
| CDS | 4392 | | |
| Pesudo gene | 8 | | |
| Total contig base | Contig1(3,181,793), Contig2(1,868,114) | | |

**TALBE S5.** VF gene profiles of all isolates

| No. | VF gene number | ARG number |
| --- | --- | --- |
| S1 | 532 | 5 |
| S2 | 539 | 5 |
| S3 | 550 | 6 |
| S4 | 536 | 5 |
| S5 | 550 | 6 |
| S6 | 540 | 5 |
| S7 | 535 | 5 |
| S8 | 542 | 5 |
| S9 | 545 | 5 |
| S10 | 547 | 5 |
| S11 | 541 | 5 |
| S12 | 672 | 5 |
| S13 | 683 | 5 |
| S14 | 533 | 7 |
| S15 | 536 | 5 |
| S16 | 531 | 5 |
| S17 | 534 | 5 |
| S18 | 540 | 5 |
| S19 | 532 | 5 |
| S20 | 539 | 5 |
| S21 | 535 | 5 |

**References**

[1] C.A. Kaysner, DePaola, A, Vibrio. Bacteriological Analytical Manual Online., U.S. Food and Drug Administration, (2004).

[2] E. Sanjuan, C. Amaro, Multiplex PCR assay for detection of Vibrio vulnificus biotype 2 and simultaneous discrimination of serovar E strains, Applied and environmental microbiology, 73 (2007) 2029-2032.

[3] E.B. Warner, J.D. Oliver, Multiplex PCR Assay for Detection and Simultaneous Differentiation of Genotypes of Vibrio vulnificus Biotype 1, Foodborne Pathogens and Disease, 5 (2008) 691-693.

[4] E. Warner, J.D. Oliver, Population structures of two genotypes of Vibrio vulnificus in oysters (Crassostrea virginica) and seawater, Applied and environmental microbiology, 74 (2008) 80-85.

[5] C.T. Lee, C. Amaro, E. Sanjuán, L.I. Hor, Identification of DNA sequences specific for Vibrio vulnificus biotype 2 strains by suppression subtractive hybridization, Applied and environmental microbiology, 71 (2005) 5593-5597.
